# Scrub Typhus Involving Central Nervous System, India, 2004–2006

**DOI:** 10.3201/eid1610.100456

**Published:** 2010-10

**Authors:** Sanjay K. Mahajan, Jean-Marc Rolain, Anil Kanga, Didier Raoult

**Affiliations:** Author affiliations: Indira Gandhi Medical College, Shimla, India (S.K. Mahajan, A. Kanga);; Université de la Méditerranée, Marseille, France (J.-M. Rolain, D. Raoult)

**Keywords:** vector-borne infections, bacteria, typhus, rickettsia, zoonoses, India, letter

**To the Editor:** Scrub typhus, caused by *Orientia tsutsugamushi,* is one of the most common infectious diseases of rural southern Asia, southeastern Asia, and the western Pacific. The disease is transmitted to humans by the bite of larvae of trombiculid mites harboring the pathogen. The disease often appears as a nonspecific febrile illness. The clinical picture of scrub typhus is typically associated with fever, rash, myalgia, and diffuse lymphadenopathy (*1*). Immunofluorescence assay (IFA) is the test of choice for serodiagnosis of rickettsial diseases (*2*). Scrub typhus has been reported from northern, eastern, and southern India, and its presence has been documented in at least 11 Indian states (*3**–**7*).

Our study’s goal was to retrospectively analyze data of patients with scrub typhus involving the central nervous system. Scrub typhus was suspected on the basis of clinical signs such as febrile illness or fever with rash or eschar. The fever workup profile (Widal agglutination test, peripheral smear, blood, and urine culture) was noncontributory. Blood samples were obtained after patients gave informed consent. All patients with clinically suspected scrub typhus received antirickettsial drugs (doxycycline and/or azithromycin) empirically. IFA and PCR of blood samples were performed to confirm scrub typhus following standard protocol (*3*). DNA was extracted from the blood sample (buffy coat) by using QIAamp DNA Mini Kit (QIAGEN GmbH, Hilden, Germany) according to the manufacturer’s instructions. A standard PCR specific for the 56-kDa protein with forward and reverse primers (OtsuF: 5′-AATTGCTAGTGCAATGTCTG-3′ and OtsuR: 5′-GGCATTATAGTAGGCTGAG-3′) was performed (*3*). PCR products were purified by using the QIAquick PCR Purification Kit (QIAGEN) according to the manufacturer’s instructions. Sequencing reactions were done by using a DNA sequencing kit, dRhodamine Terminator Cycle Sequencing Ready Reaction Mix (Applied Biosystems, Foster City, CA, USA). Sequencing was performed on an ABI PRISM 310 DNA Sequencer (Applied Biosystems). The obtained sequences were identified by comparison with sequences available in GenBank by using the BLAST software (http://blast.ncbi.nlm.nih.gov) (*3*).

During 2004–2006, scrub typhus was confirmed in 27 patients; 4 had features of central nervous system involvement. All 4 had fever with altered sensorium and meningeal signs; 2 had seizures. No neurologic focal deficit was noted, but all showed cerebrospinal fluid abnormalities. One patient had an eschar, but none had a rash. Serum of 2 patients was subjected to IFA; both samples showed high titers (Table), and PCR for blood was positive for *O. tsutsugamushi* for all patients. Serum was not subjected to examination for leptospirosis. Patients were treated mainly on the basis of clinical grounds because results of serology were not available immediately. Some clinical features of scrub typhus and leptospirosis are similar, and dual infections have been reported (*8*); therefore, antimicrobial drugs active against both leptospirosis and scrub typhus were included in treatment regimens. One patient received doxycycline and azithromycin, and the remaining 3 received ceftriaxone in addition to doxycycline. Two patients improved, 1 died, and 1 left hospital against medical advice. The clinical and laboratory details, treatments, and outcomes of all patients are given in the Table.

**Table:** Clinical features and laboratory investigations of patients who had scrub typhus with central nervous system involvement, India, 2004–2006*

Clinical and laboratory features	Results			
	Patient 1	Patient 2	Patient 3	Patient 4
Age, y/sex	52/M	50/F	30/F	47/F
Date of hospital admission	2004 Aug 22	2004 Aug 30	2005 Sep 9	2005 Sep 15
Fever duration before admission, d†	12	12	9	16
Chills	+	+	+	−
Rigors	+	+	+	−
Headache	+	+	+	−
Myalgia	+	+	+	+
Abdominal pain	+	+	−	−
Seizure	−	+	+	−
Altered sensorium	+	+	+	+
Conjunctival suffusion	+	−	+	+
Jaundice	+	+	+	+
Eschar	+ Axilla	−	−	−
Lymphadenopathy	+ Generalized	+ Cervical	+ Cervical	−
Meningeal signs	+	+	+	+
Urea, mg/dL	104	96	84	143
Creatinine, mg/dL	3.9	1.5	1.6	2.7
Bilirubin, mg/dL				
Total	3.5	2.7	4.6	3.0
Conjugated	1.0	1.7	3.6	2.6
Aspartate aminotransferase, IU	167	160	166	30
Alanine aminotransferase, IU	139	198	185	38
Alkaline phosphatase, IU	80	1,000	1,000	782
Proteinuria, mg/dL	+	−	+	+
CSF cytology	Lymphocytes, 54 cells/mm^3^	Lymphocytes, 14 cells/mm^3^	Lymphocytes, 60 cells/mm^3^	Neutrophils, 38 cells/mm^3^
Protein, mg/dL	125	69	34	118
Glucose, mg/dL	34	44	33	48
IFA titers‡				
IgG	512	512	Not done	Not done
IgM	<64	<64		
Drug treatment	Azithromycin, doxycycline	Ceftriaxone, doxycycline	Ceftriaxone, doxycycline	Ceftriaxone, doxycycline
Outcome	Died	Improved	Improved	Left hospital against advice

*CSF, cerebrospinal fluid; IFA, immunofluorescence assay; Ig, immunoglobulin. †Fever defined as AM temperature >98.9^o^ F or PM temperature >99.9^o^ F. ‡IFA significant titers: IgG >128; IgM >64. The single serum samples, obtained at admission to hospital, were subjected to IFA by using a panel of 11 rickettsial antigens comprising spotted fever group rickettsiae (*Rickettsia japonica, R. helvetica, R. slovaca, R. conorii* subsp. *indica, R. honeï, R. heilongjangensis, and R. felis*), *R. typhi*, and *Orientia tsutsugamushi* (Gilliam, Kato, and Kawasaki strains).

*O. tsutsugamushi* is an obligate intracellular parasite of professional and nonprofessional phagocytes that invades the central nervous system as part of systemic infection and is found in endothelial cells of blood vessels and in circulating phagocytes. A severe headache occurs almost invariably and has been used as a key clinical criterion for identifying suspected cases. Severe features of central nervous system involvement, such as neck stiffness, neurologic weakness, seizures, delirium, and coma, have been reported. Meningismus or meningitis has been found in 5.7%–13.5% of patients (*9*). The greatest degree of central nervous system involvement in rickettsial diseases occurs in Rocky Mountain spotted fever and epidemic typhus, followed closely by scrub typhus. The meninges are more commonly involved by *O. tsutsugamushi* than by other rickettsial infections, and the overall histologic picture in the central nervous system is best described as a meningoencephalitis (*9*). An exhaustive study of 200 cases of scrub typhus showed central nervous system involvement in most patients. However, focal central nervous system damage was rare, and during the encephalitis stage, few objective neurologic signs were apparent, other than those suggesting more generalized cerebral involvement, such as confusion, tremor, and restlessness (*10*).

Now that it is established that *O. tsutsugamushi* does invade cerebrospinal fluid, scrub typhus should be considered a cause of mononuclear meningitis in areas in which it is endemic. In our study 1 patient died despite treatment with doxycycline and azithromycin, suggesting the possibility of resistance to these antimicrobial drugs as recently posited in a study conducted in southern India (*6*). Scrub typhus in these regions should be further investigated in prospective studies, and clinical isolates should be obtained to evaluate susceptibility to antimicrobial drugs.
